# First Ex Vivo Animal Study of a Biological Heart Valve Prosthesis Sensorized with Intravalvular Impedance

**DOI:** 10.3390/s23083829

**Published:** 2023-04-08

**Authors:** Laura Cercenelli, Camilla Gironi, Barbara Bortolani, Emanuela Marcelli

**Affiliations:** Laboratory of Bioengineering—eDIMES Lab, Department of Medical and Surgical Sciences (DIMEC), University of Bologna, 40138 Bologna, Italy; laura.cercenelli@unibo.it (L.C.); barbara.bortolani@unibo.it (B.B.); emanuela.marcelli@unibo.it (E.M.)

**Keywords:** heart valve prosthesis, electric impedance, implantable sensor, cardiac biosimulator, ex vivo animal experiments, cardiac monitoring

## Abstract

IntraValvular Impedance (IVI) sensing is an innovative concept for monitoring heart valve prostheses after implant. We recently demonstrated IVI sensing feasible in vitro for biological heart valves (BHVs). In this study, for the first time, we investigate ex vivo the IVI sensing applied to a BHV when it is surrounded by biological tissue, similar to a real implant condition. A commercial model of BHV was sensorized with three miniaturized electrodes embedded in the commissures of the valve leaflets and connected to an external impedance measurement unit. To perform ex vivo animal tests, the sensorized BHV was implanted in the aortic position of an explanted porcine heart, which was connected to a cardiac BioSimulator platform. The IVI signal was recorded in different dynamic cardiac conditions reproduced with the BioSimulator, varying the cardiac cycle rate and the stroke volume. For each condition, the maximum percent variation in the IVI signal was evaluated and compared. The IVI signal was also processed to calculate its first derivative (dIVI/dt), which should reflect the rate of the valve leaflets opening/closing. The results demonstrated that the IVI signal is well detectable when the sensorized BHV is surrounded by biological tissue, maintaining the similar increasing/decreasing trend that was found during in vitro experiments. The signal can also be informative on the rate of valve opening/closing, as indicated by the changes in dIVI/dt in different dynamic cardiac conditions.

## 1. Introduction

Surgical Leaflet Thrombosis (SLT) occurs in 5% to 40% of patients undergoing surgical or transcatheter replacement of the aortic heart valve prosthesis (HVP) [1,2,3,4,5,6,7,8]. The incidence of SLT is highly influenced by the timing of screening and the imaging tools used to detect it in the clinical practice. Multidetector computed tomography is the gold-standard tool for the diagnosis of SLT, since it allows one to accurately detect both the hypoattenuating leaflet thrombosis and the reduced leaflet motion which characterize SLT, although its routine use in clinical practice is not recommended [6,8,9,10,11]. Moreover, several studies have suggested anticoagulant therapy as an optimal strategy to prevent and reduce the occurrence of SLT formation, suggesting the need for early detection and a subsequent tailored therapy following valve replacement [9,11,12,13,14].

An alternative approach to using imaging for monitoring the HVP functionality after implantation may be represented by the use of sensing means of the valve prosthesis itself. Previous attempts have been made for the integration of piezo-electric sensors to the prosthetic structure to evaluate the valve functionality by exploiting a time–frequency analysis of the acquired vibro-acoustic signals [15]. A recent study coupled signal processing with machine learning for the evaluation of the mobility recorded by a miniaturized pressure sensor embedded in the prosthetic valve structure [16]. Other studies proposed the use of magnetic sensors embedded in the valve leaflets for the quantification of the transvalvular flow and for the monitoring of leaflets’ movements [17,18].

In this regard, we recently proposed a novel sensing approach for HVPs, which we call “IntraValvular Impedance” (IVI), based on using miniaturized electrodes for an electric impedance measurement [19]. The IVI sensing was first evaluated on multiple proof-of-concept prototypes of sensorized mechanical heart valves (MHVs) that we designed and tested in circulatory mock loops [20]. We subsequently presented the conceptual design of the IVI measurement applied to biological heart valves (BHVs) [21]. In particular, we compared different solutions for the electrodes embedded in the BHV in terms of size, shape and positioning, and we tested them in vitro on a circulatory mock loop reproducing both normal and altered dynamics of the valve leaflets. These analyses allowed us to identify the optimal electrode configuration, i.e., the one characterized by a higher sensitivity of the impedance signal to experimentally induced changes in the leaflet motions. This optimal configuration was represented by small parallelepiped-shaped electrodes embedded in the commissures of the BHV [21].

As a next step of investigation, it would be very interesting to evaluate the IVI signal variation when the BHV is surrounded by biological tissue, similar to what happens in vivo. Indeed, this configuration was never reproduced in our previous studies where the sensorized valve was included in a customized polymeric housing and tested on a circulatory mock loop system [21].

The present study describes the first ex vivo evaluation of the novel IVI sensing approach applied to BHVs, i.e., with the sensorized BHV implanted inside an explanted porcine heart connected to a cardiac BioSimulator platform.

## 2. Materials and Methods

### 2.1. IVI Sensing Applied to a BHV

For the study, we applied the IVI sensing to a commercial BHV currently used for surgical aortic valve replacement (Soprano Armonia, LivaNova PCL, London, UK).

As we previously described [20,21], the IVI sensing is based on embedding miniaturized electrodes in the structure of the prosthetic valve, which are used for both the local electric field generation (current injection, I) and recording of electric potential difference (∆V). Following Ohm’s first law, the impedance measurement (IVI) is obtained as the ratio between the recorded ∆V over the injected I. Since the valve leaflets interfere with the local electric field lines during the valve opening and closing dynamics, IVI variations within the cardiac cycle (ΔIVI) reflect the cyclic movement of the valve leaflets and its possible alterations due to the presence of an obstacle, such as thrombus formations.

**Figure 1:**
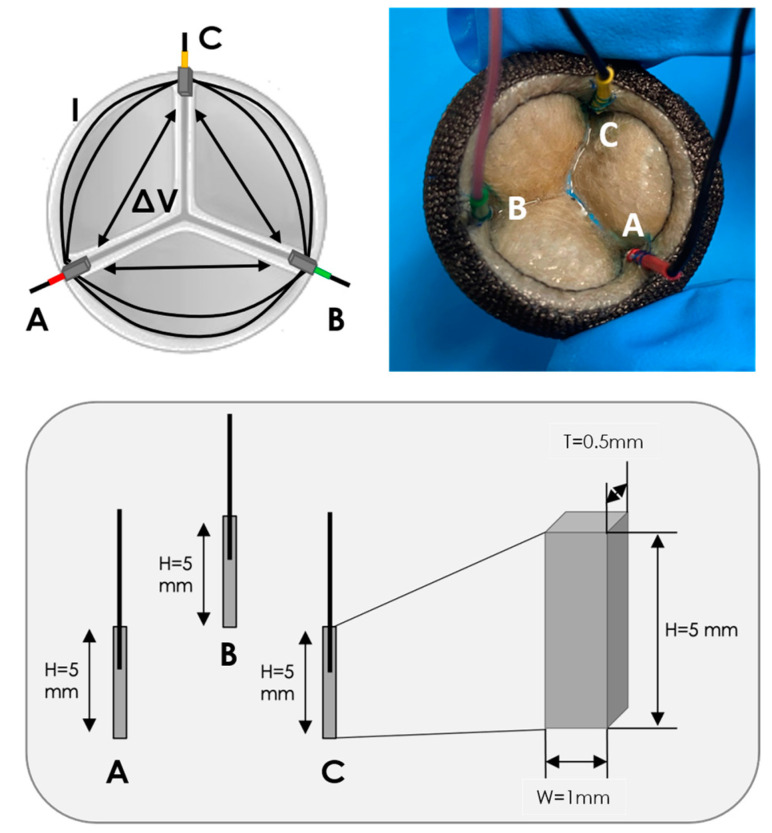
Small parallelepiped-shaped electrodes (A, B, C) embedded in the commissures of the BHV.

The selected aortic BHV was sensorized with three electrodes, conventionally called “A”, “B” and “C”, following the optimal electrode configuration we previously identified [21]. These electrodes have a parallelepiped shape (height H = 5 mm, width W = 1 mm, thickness T = 0.5 mm) and are manufactured in Pt/Ir, which is a biocompatible alloy typically used for implantable electrodes [22] (Figure 1). The electrodes were positioned in the commissures of the BHV leaflets and held in place by sewing them to the valve structure using a suture thread. A thin conductor wire was welded to each electrode in the longitudinal direction for the connection to the external impedance measurement unit. A different color heat shrink was used to cover each electrode–wire interface and to make the electrodes distinguishable from one another: red for electrode “A”, green for electrode “B” and yellow for electrode “C” (Figure 1).

For each pair of electrodes (“AB”, “BC” and “CA”), an IVI measurement was performed according to the bipolar impedance measurement configuration, which consists of using the same pair of electrodes for both the local electric field generation by injection of current I (source electrodes) and the ΔV recording (receiver electrodes).

### 2.2. Impedance Measurement Unit

**Figure 2:**
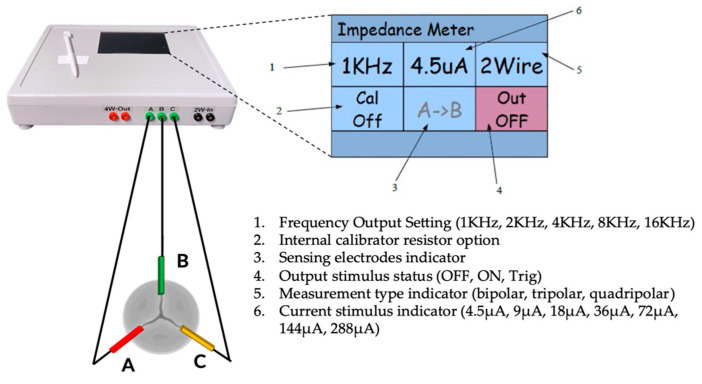
The impedance meter connected to the electrodes (A, B, C) of the sensorized valve and its settings.

The conductor wires welded to the electrodes were connected to a dedicated external impedance measurement unit (“impedance-meter”), which can be programmable and highly configurable in the settings of current amplitude and frequency, as shown in Figure 2. The impedance meter was manufactured for this specific application by a specialized company (El Radio di Enrico Lenzi, Minerbio, BO, Italy), taking as reference the Impact Custom Model 2364 (Medtronic, Minneapolis, MA, USA), which was a commercial impedance meter intended to be used with standard cardiac leads.

For all the experiments, the impedance measurements were performed by setting the measurement in “bipolar configuration” and delivering current pulses of 18 μA at 4 kHz to each of the three electrodes embedded in the BHV, thus generating a local electric field near the valve leaflets. Then, the impedance meter records the potential difference between each pair of electrodes ($\Delta V_{AB},\;\;\Delta V_{BC}$ and $\;\Delta V_{CA}$). Hence, three IVI signals are obtained ($IVI_{AB},\;\;IVI_{BC}\;$ and $\;IVI_{CA})$, one for each pair of electrodes. The calibration procedure of the impedance meter was conducted prior to each test, as previously described [21].

### 2.3. Ex Vivo Animal Testing

#### 2.3.1. Cardiac BioSimulator Platform

**Figure 3:**
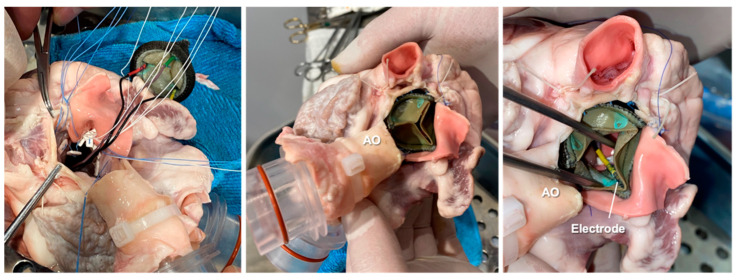
Surgical implantation of the sensorized BHV inside the explanted porcine heart. AO = aorta.

As a first step, the sensorized BHV was implanted in the aortic position of an explanted porcine heart by a specialized cardiac surgeon (Figure 3).

**Figure 4:**
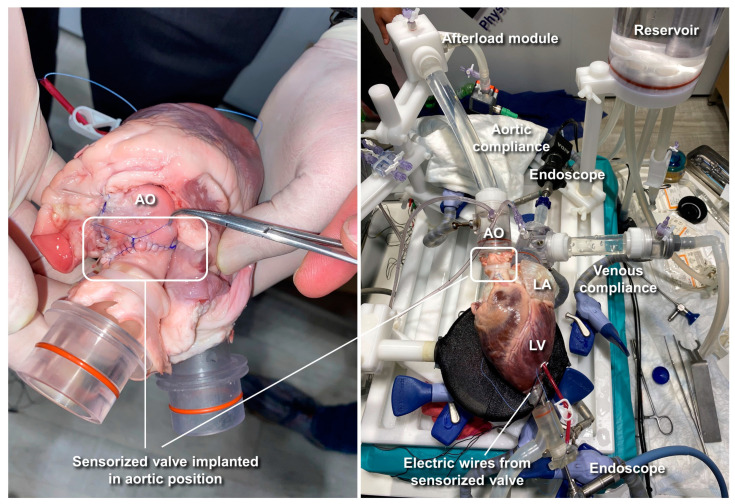
Cardiac BioSimulator platform used for the ex vivo animal testing of the sensorized BHV. AO = aorta; LA = left atrium; LV = left ventricle.

Then, the excised heart, including the sensorized prototype, was mounted on the Cardiac BioSimulator platform manufactured by LifeTec Group™ (Eindhoven, The Netherlands), as shown in Figure 4.

The Cardiac BioSimulator platform consists of an explanted porcine heart to which a pulsatile fluid dynamic system is connected [23]. As shown in Figure 4, a piston is attached to the apex of the left ventricle (LV) and allows one to pressurize the cardiac chamber and to replicate the pulsatile flow. The working fluid is NaCl 0.9% in aqueous solution which approximates the electrical conductivity of the blood. All experiments were conducted at room temperature (~22 °C), so the working fluid inside the ex vivo platform was maintained at room temperature.

The ascending aorta (AO) is connected to a compliant silicone tube that mimics the aortic compliance and to an afterload module that simulates peripheral vessel resistance. The working fluid then passes through a reservoir and afterwards it is taken by a centrifugal pump to fill the left atrium (LA). Before reaching the LA, the fluid passes through a second compliant silicone tube that mimics the venous compliance and an adjustable Starling resistor that allows atrium pressure to be maintained to physiological values. The platform is equipped with two intracardiac endoscopes that are passed into the LA and LV to perform video recording of the mitral and aortic valves, respectively. In particular, the LV endoscope allowed us to monitor the implanted sensorized BHV during its functioning (Figure 4, Supplementary Video S1).

Two pressure sensors (P10-EX, Becton Dickinson, NJ, USA) connected to the circulatory loop allowed us to measure the left atrial and aortic pressure during the tests. The implanted electrode wires were brought out of the LV through a small ventriculostomy and then secured to the epicardium using a plastic anchor (Figure 4). IVI measurement was performed connecting the emerging wires to the impedance meter.

The analog signals (left atrial pressure, aortic pressure and IVI) were acquired with the MP100 Acquisition System (Biopac System, Galeta, CA, USA) and were displayed in real time on a PC using Acq3.9.1 Software (Biopac System, Galeta, CA, USA).

#### 2.3.2. Test Conditions

We evaluated the IVI measurement under different working conditions by varying the cardiac cycle rate and stroke volume parameters in the Cardiac BioSimulator platform as follows:

- Test 1: cycle rate 40 bpm, stroke volume 70 mL;
- Test 2: cycle rate 50 bpm, stroke volume 70 mL;
- Test 3: cycle rate 50 bpm, stroke volume 80 mL.

For all the experiments, the reproduced aortic and LA pressures were in the ranges of 10–60 mmHg and 0–10 mmHg, respectively.

For each test, the IVI signal was recorded between the three pairs of electrodes, and IVI variations during the fully opening/closing dynamics of the valve were compared.

### 2.4. Data Analysis and Statistics

We evaluated the maximum percent variation in the impedance module ($\Delta IVI_{max}\%$), i.e., the maximum excursion of IVI measurement within the cardiac cycle. $\Delta IVI_{max}\%$ is calculated as the difference between the maximum and minimum value reached during the opening/closing dynamics, normalized with respect to the minimum value reached during each opening/closing, as shown in Equation (1).

(1) $$\Delta IVI_{max}\%=\frac{\left|IVI_{max}-IVI_{min}\right|}{IVI_{min}}*100$$

The acquired analog signals were post-processed with MATLAB (R2019a, MathWorks, Natick, MA, USA). A low-pass filter (cut-off frequency 5 Hz) was applied to the IVI signals in order to reduce the noise component. This cut-off frequency was empirically determined as the one capable of removing the high-frequency oscillations due to the vibrations induced by the circuit pumps and to the electronic noise induced into the IVI signal traveling the cable.

The low peaks of aortic pressure signal were used to identify the opening time (“O”) of the valve at each cardiac cycle, and it was not possible to determine the closing time of the valve since the left ventricular pressure signal was not acquired during the tests.

For each experimental condition, ${\Delta \mathrm{IVI}}_{\max}\%$ was reported as mean value±SD, calculated over 16 cardiac cycles.

We also processed the recorded IVI signal in order to calculate its first derivative (dIVI/dt), which should reflect the rate of the opening/closing of the valve leaflets. From the dIVI/dt signal, the negative peak, corresponding to the highest opening Rate ($oR_{max}$), was calculated and averaged over 16 cardiac cycles.

For comparative evaluation between the different testing conditions, statistical analysis was performed using Student’s *t*-test. A *p*-value of 0.05 was chosen as significant. All analyses were made with SPSS version 23.0 (IBM SPSS, New York, NY, USA).

## 3. Results

**Figure 5:**
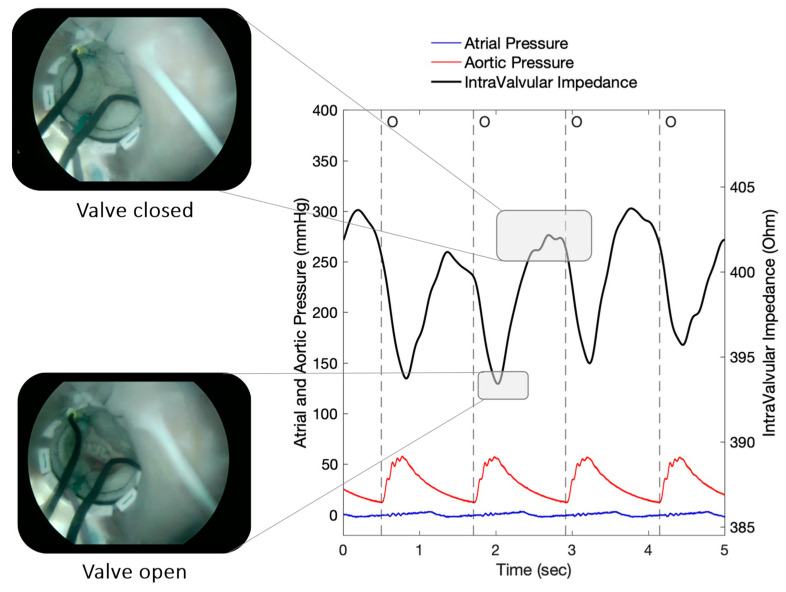
IVI signal profile reflecting the opening and closing dynamics of the valve. O = start of valve opening identified with the low peak of the aortic pressure.

An example of the recorded IVI signal from the implanted sensorized BHV is shown in Figure 5. The signal reflects the opening/closing dynamics of the valve leaflets, with maximum values corresponding to complete valve closing and minimum values corresponding to valve opening, as demonstrated by the simultaneous endoscopic video recordings (Figure 5).

**Figure 6:**
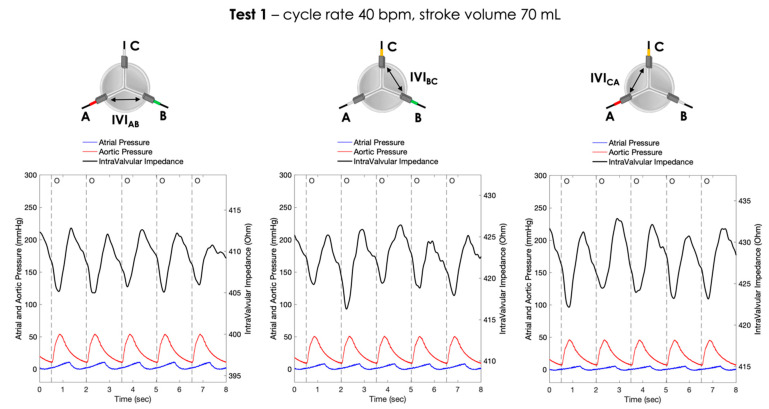
Atrial pressure, aortic pressure and IVI signal recorded inside the porcine heart for the three pairs of electrodes during Test 1 (cycle rate 40 bpm, stroke volume 70 mL): AB (**left**), BC (**center**) and CA (**right**). O = start of valve opening identified with the low peak of the aortic pressure.

**Figure 7:**
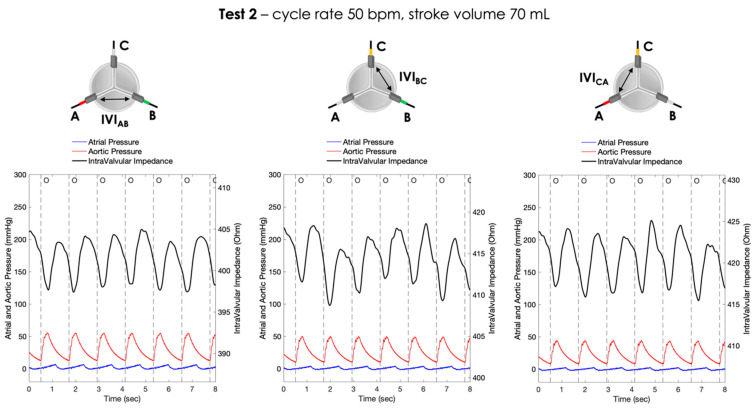
Atrial pressure, aortic pressure and IVI signal recorded inside the porcine heart in the three pairs of electrodes during Test 2 (cycle rate 50 bpm, stroke volume 70 mL): AB (**left**), BC (**center**) and CA (**right**). O = start of valve opening identified with the low peak of the aortic pressure.

**Figure 8:**
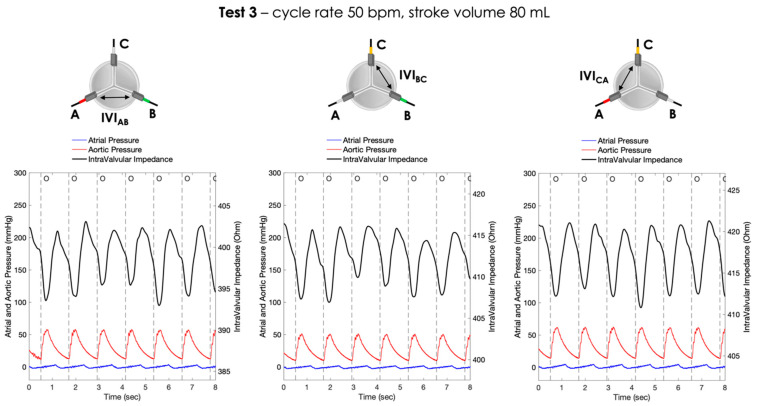
Atrial and aortic pressures and IVI signal recorded inside the porcine heart in the three pairs of electrodes during Test 3 (cycle rate 50 bpm, stroke volume 80 mL): AB (**left**), BC (**center**) and CA (**right**). O = start of valve opening identified with the low peak of the aortic pressure.

For each pair of electrodes, the pressure signals and the IVI signals recorded under Test 1, Test 2 and Test 3 working conditions are shown in Figure 6, Figure 7 and Figure 8, respectively.

For each working condition, the maximum percent variation in the impedance module (${\Delta \mathrm{IVI}}_{\max}\%$), evaluated for each pair of electrodes, was then calculated following Equation (1) as shown in Table 1.

In Test 1, the maximum percent variation in the IVI module was obtained in the electrode pair CA (1.88±0.23%), followed by AB (1.68±0.24%) and BC (1.65±0.24%).

In Test 2, the maximum percent variation in the IVI module was obtained in the electrode pair CA (1.94±0.19%), followed by BC (1.74±0.24%) and AB (1.69±0.17%).

In Test 3, the maximum percent variation in the IVI module was obtained in the electrode pair CA (2.13±0.18%), followed by AB (2.08±0.27%) and BC (1.81±0.21%).

However, only some of these differences were statistically significant (Table 2).

**Figure 9:**
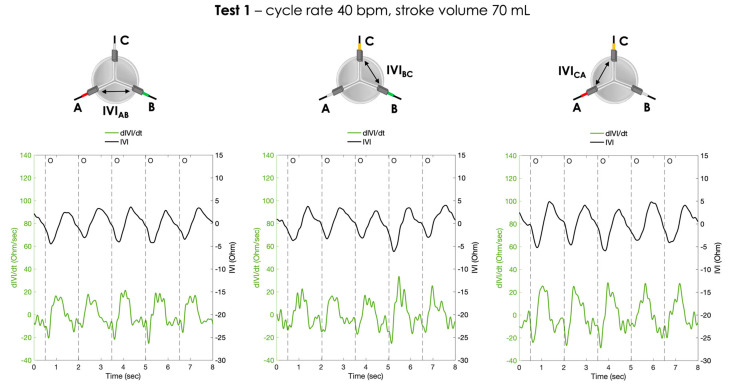
The IVI signal (black line) and the corresponding first derivative dIVI/dt (green line) obtained for the three pairs of electrodes during Test 1 (cycle rate 40 bpm, stroke volume 70 mL). AB (**left**), BC (**center**) and CA (**right**). O = start of valve opening identified with the low peak of the aortic pressure.

**Figure 10:**
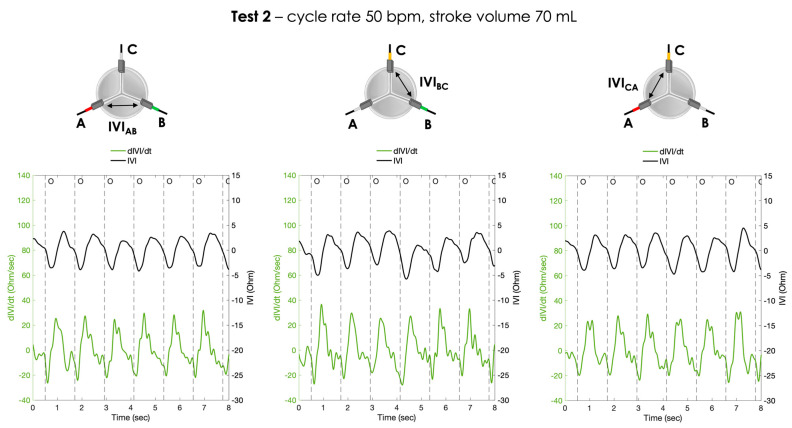
The IVI signal (black line) and the corresponding first derivative dIVI/dt (green line) obtained for the three pairs of electrodes during Test 2 (cycle rate 50 bpm, stroke volume 70 mL). AB (**left**), BC (**center**) and CA (**right**). O = start of valve opening identified with the low peak of the aortic pressure.

**Figure 11:**
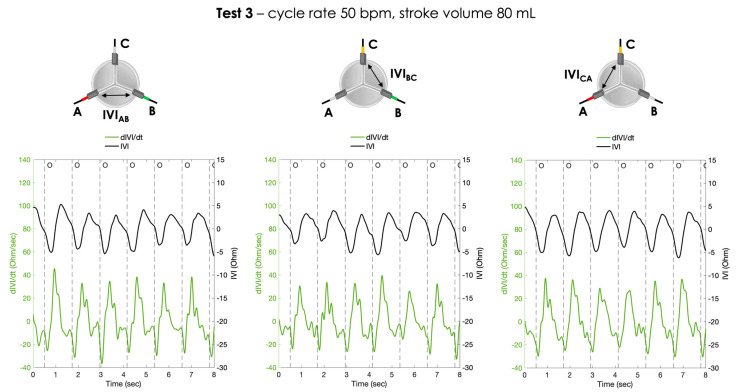
The IVI signal (black line) and the corresponding first derivative dIVI/dt (green line) obtained for the three pairs of electrodes during Test 3 (cycle rate 50 bpm, stroke volume 80 mL). AB (**left**), BC (**center**) and CA (**right**). O = start of valve opening identified with the low peak of the aortic pressure.

The calculated first derivative of the IVI signal (dIVI/dt) was reported for the three testing conditions, for each pair of electrodes, in Figure 9, Figure 10 and Figure 11.

For each working condition, the maximum opening rate (oR__max_) represented by the negative peak of dIVI/dt curve was calculated and reported for each pair of electrodes in Table 3. The results obtained for the comparative analysis between the various test conditions in terms of statistically significant difference were summarized in Table 4.

**Figure 12:**
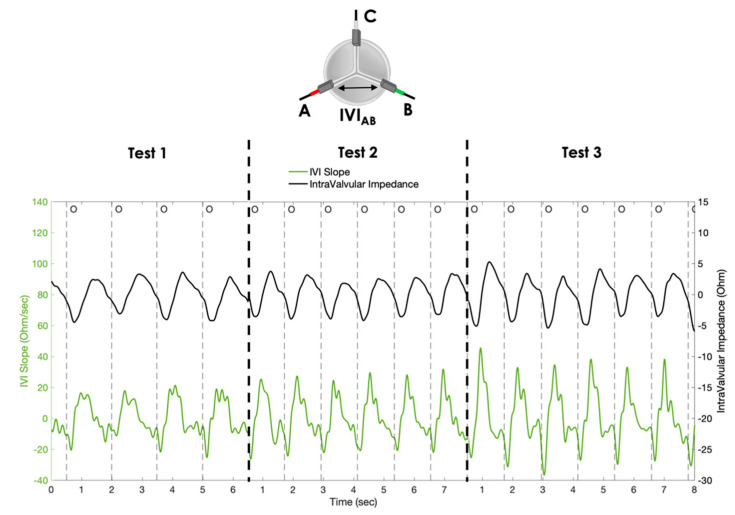
IVI signal (black line) and the corresponding dIVI/dt (green line) obtained with the AB pair of electrodes for the three different testing conditions (Test 1, Test 2, Test 3). O = start of valve opening identified with the low peak of the aortic pressure.

Finally, for comparative purposes, the IVI signal and the corresponding dIVI/dt signal, obtained with the AB pair of electrodes (as an example) for the three simulated conditions, are reported in the same graph (Figure 12).

## 4. Discussion

This study aimed to investigate the IVI measurement applied to a sensorized BHV when positioned in a biological environment similar to the real one. This condition was reproduced by surgically implanting the prototype in the aortic position of an explanted porcine heart, implemented into a cardiac BioSimulator platform, which ensures a realistic pulsatile flow through the valve.

For all the working conditions that we reproduced ex vivo, the results confirmed the presence of a detectable variation in the IVI signal module within the cardiac cycle following the dynamics of the valve. The recorded wave pattern for IVI signal was consistent with our previous findings dealing with vitro testing of a similar sensorized prototype [10]. This wave pattern can be explained by the chosen electrodes’ configuration for the IVI signal. Indeed, the maximum IVI signal can be observed when the valve is closed, as the valve leaflets close around the electrodes, maximally interfering with the local electric field lines. On the contrary, when the valve is open, the IVI signal reaches minimum values, as the valve leaflets stretch outward, minimally interfering with the electric field (Figure 5).

**Figure 13:**
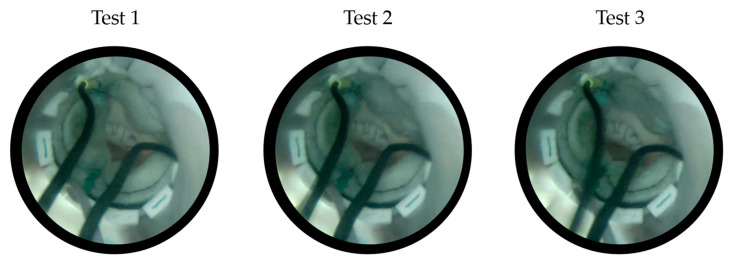
Increased opening area of the valve leaflets when passing from Test 1, Test 2, Test 3, as detected from the endoscopic images.

In this study, multiple tests were carried out by varying the parameters of the cardiac BioSimulator platform (i.e., cycle rate and stroke volume) in order to obtain different opening/closing dynamics of the valve leaflets. We observed an increasing trend in the $\Delta IVI_{max}\%\;$when increasing the cycle rate and the stroke volume, i.e., when passing from Test 1 to Test 2 and Test 3 (Table 1). In particular, the differences in IVI excursions were found statistically significant when mainly changing the stroke volume (i.e., passing from Test 1 to Test 3, and from Test 2 to Test 3). The possible explanation is that higher stroke volume may contribute to obtaining wider opening/closing dynamics of the valve leaflets, which therefore determines a higher excursion of the IVI signal due to a major interference of the leaflets with the local electric field lines during the cardiac cycle. In Test 3, this effect of wider opening/closing dynamics of the valve can also be further amplified by the simultaneous increase in cycle rate. Indeed, this increasing trend in IVI signal excursion from Test 1 to Test 3 (i.e., when both the stroke volume and the cycle rate are increased) was consistent with the “wider” dynamics of the valve leaflets that can be appreciated from the recorded endoscopic images (Figure 13).

The percentage of maximum IVI excursion (${\Delta \mathrm{IVI}}_{\max}\%$) we obtained is in line with what we previously recorded using polymeric housing for the valve in the in vitro testing platform; indeed, in that case, the ${\Delta \mathrm{IVI}}_{\max}\%$ obtained for the complete opening/closing dynamics of the valve were in the range of 2.29–3.20% for the three pairs of electrodes [21]. These findings are extremely important as they ensure that a good impedance signal can be detected even in a realistic implant condition resembling the ex vivo environment. This reasonably dispels the doubt that in the previous in vitro tests, the insertion of the sensorized valve in Plexiglas housing with potentially very reflective walls for the electric field might have induced an amplified IVI signal response.

The IVI measurement shows slight variability between the three pairs of electrodes $(IVI_{AB},\;IVI_{BC}\;$ and $\;IVI_{CA})$, which can be mainly related to the manual positioning and sewing of the electrode in each commissure, besides the intrinsic variability of the heartbeat and the natural asymmetric movements of the leaflets.

Regarding the dIVI/dt signal, we observed that it can be informative on the rate of valve opening/closing, as indicated by the significant increase in oR__max_ as both the cycle rate and stroke volume increase. This means that when the valve opening is wider and faster (i.e., passing from Test 1 to Test 3), the slope of IVI variation between the complete closure and maximum opening becomes steeper (Figure 12).

### Study Limitations and Future Directions

One limitation of the study is related to the used working fluid, i.e., a saline solution, which only approximates the electrical conductivity of the blood [20]. Data from the National Institute of Standards and Technology indicate a conductivity of κ = 14.5 mS/cm for a 0.9% saline solution at 22 °C, which corresponded to a resistance of 76 Mohm. Indeed, the blood conductivity is affected by temperature, red blood cells and plasma components, and all these effects are not considered when using the saline solution as a working fluid. Moreover, the saline solution does not replicate the blood viscosity, especially given the fact that the blood is a non-Newtonian fluid.

Surely, future in vivo animal experiments are needed to investigate the effect of warm clottable blood on the IVI signal. The in vivo implantation will be fundamental also to evaluate how the IVI sensing accuracy may be affected by a foreign body reaction caused by the sensor in contact with blood, such as clot or biofilm formation.

Another limitation is that the cardiac BioSimulator platform was not provided with a flow sensor to also detect the transvalvular flow during the acquisitions. Moreover, the BioSimulator platform was not provided with the LV pressure sensing, which may be useful to determine the closing time of the valve within the cardiac cycle.

In our ex vivo animal experiments, the selected heart was slightly too small compared to the size of the implanted sensorized prosthesis. This could have caused an incomplete opening of the leaflets, as the valve was somewhat forced inside the aortic tract and therefore impeded to achieve a complete opening (Figure 13). This may have limited the maximum detectable IVI signal excursion between the closed and open condition.

Moreover, the implantation of a too large heart valve prosthesis for the excised heart used in the platform has implied to set “reduced” opening/closing dynamics (quite low heart rates and perfusion pressures) compared to the physiological values for adult hearts, so as to avoid to excessively stressing the implant site, which is somewhat precarious due to this mismatch between the heart valve size and the animal valvular annulus. Future research should aim to replicate these experiments in a wider range of working conditions, such as performing a hemodynamically altered situation in which the valve leaflets do not fully close due to the reduced mobility induced by a simulated “hypotensive” pressure condition. Another experiment could focus on reproducing an altered condition only for one leaflet, supposing to have a thrombus formation that mostly impedes the movement of this leaflet. In this case, there should be a difference in the IVI signals recorded between the different pairs of electrodes, with a reduction in excursion for those pairs involving electrodes positioned in the commissures of the altered leaflet.

In addition, further experiments could be planned to investigate if different values of current pulse amplitude or frequency may determine any significant variation in the detected IVI signal.

Alongside these experimental evaluations, it would be extremely useful to develop and validate an in silico simulation tool that reproduces this principle of IVI sensing applied to different BHV models, using, for example, dedicated toolboxes for electric field simulation available in Ansys and/or COMSOL software. This may help to further optimize the electrode size, shape and positioning according to different prosthesis models, as well as to simulate various altered working conditions for the leaflet, also including thrombus formation.

Finally, further studies will be necessary to provide an implantable prototype compatible with a real situation, i.e., not requiring the current wired connections. This wireless solution will have to include a miniaturized Application-Specific Integrated Circuit (ASIC) on board the prosthesis and means for the powering and telemetric communication of the acquired IVI signal, via an external unit [21].

## 5. Conclusions

The present study reports the first ex vivo evaluation of the novel IVI measurement applied to a BHV to demonstrate that the new concept can also work in conditions very similar to the in vivo one.

The paper brings an important advantage in that it demonstrates that the IVI signal is detectable also when the sensorized BHV is surrounded by biological tissue, and the signal increase/decrease trend is in agreement with the in vitro experiments.

Another remarkable result of the paper is that it identifies the first-order derivative of the IVI signal with respect to time as a measure of the opening/closing rate of the sensorized biological heart valve prosthesis, for different dynamic operating regimes.

All these findings are encouraging to plan future in vivo animal evaluations and to further pursue the development of a fully implantable wireless solution of IVI-sensorized BHVs.

## 6. Patents

From the work reported in this manuscript, the following issued patents result:

- WO2015EP58201 20150415. Heart valve prosthesis with integrated electronic circuit for measuring intravalvular electrical impedance, and system for monitoring functionality of the prosthesis. E. Marcelli (Inventor); Alma Mater Studiorum (Applicant). Filed: 15 April 2015.
- Also published as: EP3131502 (A1); CN106456043 (A); US9987129 (B2)—Issued: 5 June 2018.
- N. 0001423344 Protesi valvolare cardiaca con circuito elettronico integrato per effettuare misure di impedenza elettrica intravalvolare e sistema per monitorare la funzionalità di tale protesi—E. Marcelli (Inventor); Alma Mater Studiorum (Applicant). Filed: 16 April 2014. Issued: 22 July 2016.

## Figures and Tables

**Table 1:** Maximum percent variations in the impedance module, reported as $\Delta IVI_{max}\%\pm \mathrm{SD}$ calculated over 16 cardiac cycles, for each pair of electrodes (AB, BC and CA) in the three testing conditions (Test 1, Test 2, Test 3).

	Test 1 (Mean ± SD)	Test 2 (Mean ± SD)	Test 3 (Mean ± SD)
$\Delta IVI_{AB,max}\%$	1.68±0.24	1.69±0.17	2.08±0.27
$\Delta IVI_{BC,max}\%$	1.65±0.24	1.74±0.24	1.81±0.21
$\Delta IVI_{CA,max}\%$	1.88±0.23	1.94±0.19	2.13±0.18

**Table 2:** *p*-value obtained from Student’s *t*-test comparing $\Delta IVI_{max}\%$ under different working conditions (Test 1, Test 2 and Test 3) for the three pairs of electrodes (AB, BC and CA). The asterisk indicates significant *p*-values.

Electrodes	Test 1 vs. Test 2	Test 2 vs. Test 3	Test 1 vs. Test 3
AB	0.42	* <0.001	* <0.001
BC	0.28	* 0.036	* 0.024
CA	0.27	* 0.001	* <0.001

**Table 3:** For each pair of electrodes (AB, BC and CA), the maximum opening rate (oR__max_) is shown for the three working conditions (Test 1, Test 2, Test 3). Data are reported as mean values ±SD calculated over 16 cardiac cycles.

	Test 1 (Mean ± SD)	Test 2 (Mean ± SD)	Test 3 (Mean ± SD)
$oR_{AB,\;max}$ [Ohm/s]	18.3±4.0	23.4±3.8	27.1±5.3
$oR_{BC,\max}$ [Ohm/s]	19.1±7.0	23.8±4.2	25.3±4.3
$oR_{CA,\;max}$ [Ohm/s]	21.7±4.4	22.6±4.3	27.8±6.2

**Table 4:** *p*-value obtained from Student’s *t*-test comparing oR__max_ under different working conditions (Test 1, Test 2 and Test 3) for the three pairs of electrodes (AB, BC and CA). The asterisk indicates significant *p*-values.

Electrodes	Test 1 vs. Test 2	Test 2 vs. Test 3	Test 1 vs. Test 3
AB	* <0.001	* 0.002	* 0.001
BC	* 0.01	0.125	* 0.001
CA	0.483	* <0.001	* 0.001

## Data Availability

The datasets generated during and/or analyzed during the current study are available from the corresponding author on reasonable request.

## Supplementary Materials

The following supporting information can be downloaded at: https://www.mdpi.com/article/10.3390/s23083829/s1, Video S1: the cardiac BioSimulator working with the implanted sensorized valve.
